# Top 100 cited articles in cardiovascular magnetic resonance: a bibliometric analysis

**DOI:** 10.1186/s12968-016-0303-9

**Published:** 2016-11-21

**Authors:** Muhammad Shahzeb Khan, Waqas Ullah, Irbaz Bin Riaz, Nizar Bhulani, Warren J. Manning, Srini Tridandapani, Faisal Khosa

**Affiliations:** 1 Department of Internal Medicine, Dow Medical College, Karachi, Pakistan; 2 Department of Internal Medicine, Khyber Medical College, Peshawar, Pakistan; 3 Department of Internal Medicine, University of Arizona, Tuscon, AZ USA; 4 Department of Medical Oncology, University of Texas, MD Anderson Cancer Center, Houston, TX USA; 5 Department of Medicine (Cardiovascular Division) and Radiology, Beth Israel Deaconess Medical Center and Harvard Medical School, Boston, MA USA; 6 Department of Radiology and Imaging Sciences, Emory University, Atlanta, GA, USA; School of Electrical and Computer Engineering, Georgia Institute of Technology, Atlanta, GA USA; 7 Radiology Department, Vancouver General Hospital, University of British Columbia, Vancouver, BC Canada

**Keywords:** Bibliometrics, Cardiac, MRI, Web of Science, Scopus

## Abstract

**Background:**

With limited health care resources, bibliometric studies can help guide researchers and research funding agencies towards areas where reallocation or increase in research activity is warranted. Bibliometric analyses have been published in many specialties and sub-specialties but our literature search did not reveal a bibliometric analysis on Cardiovascular Magnetic Resonance (CMR). The main objective of the study was to identify the trends of the top 100 cited articles on CMR research.

**Methods:**

Web of Science (WOS) search was used to create a database of all English language scientific journals. This search was then cross-referenced with a similar search term query of Scopus® to identify articles that may have been missed on the initial search. Articles were ranked by citation count and screened by two independent reviewers.

**Results:**

Citations for the top 100 articles ranged from 178 to 1925 with a median of 319.5. Only 17 articles were cited more than 500 times, and the vast majority (*n =* 72) were cited between 200–499 times. More than half of the articles (*n =* 52) were from the United States of America, and more than one quarter (*n =* 21) from the United Kingdom. More than four fifth (*n =* 86) of the articles were published between the time period 2000–2014 with only 1 article published before 1990. *Circulation* and *Journal of the American College of Cardiology* made up more than half (*n =* 62) of the list. We found 10 authors who had greater than 5 publications in the list.

**Conclusion:**

Our study provides an insight on the characteristics and quality of the most highly cited CMR literature, and a list of the most influential references related to CMR.

**Electronic supplementary material:**

The online version of this article (doi:10.1186/s12968-016-0303-9) contains supplementary material, which is available to authorized users.

## Background

Bibliometric analysis is a method to study the frequency and patterns of citations in the literature. Though it is virtually impossible to evaluate the true value of an article, citation analysis provides a simple quantitative technique to estimate the impact of an article. The role of citation frequency has long been debated; yet it remains the most commonly used tool to identify important discoveries and studies which have had a disproportionate influence in a particular field [1] Citation analysis can be an important parameter to prioritize research funding in this era emphasizing cost effectiveness. With limited health care resources, bibliometric studies can help guide researchers and research funding agencies towards areas where restriction or increase in research activity is warranted.

Cardiovascular Magnetic Resonance (CMR), a cross-sectional non-invasive method for assessment of the cardiovascular system has evolved substantially over the past 3 decades. Considering that cardiovascular disease is projected to cause 24 million deaths annually by 2030 [2], it is evident that the role of non-invasive cardiac imaging tests such as CMR will continue to grow. Bibliometric analyses have been published in many specialties and sub-specialties [3–12] but our literature search did not reveal a bibliometric analysis on CMR or cardiovascular imaging. In an attempt to bridge this gap, we conducted a citation analysis to identify the top 100 CMR articles to give cardiologists and cardiac radiologists a brief overview of landmark CMR studies.

## Methods

No Institutional Review Board approval was needed for our study as it was a retrospective evaluation of publicly available data.

Scopus Library database (www.scopus.com) was searched in March 2016 for all citations pertaining to non-invasive cardiac imaging. All the journals listed under the institute of science information web of science (WOS) subject category “Cardiovascular, cardiology and heart” were included in our study. We also searched other journals using a variety of keywords to ensure that no article was overlooked. We did not limit our search on the basis of abstract availability, study type or non-human research subjects. Time restriction was also not imposed. To maintain a relevant and focused list of CMR articles, only articles related to the field of cardiology and radiology with a primary focus on CMR were selected. All articles from journals focusing on fields of science other than medicine were excluded. All the journals were searched using both the print and electronic International Standard Serial Numbers. For articles where electronic copies were unavailable, hard copies were sought from inter-library loan service.

After an extensive search, all the retrieved articles were sorted according to the option “Times cited”. Two reviewers (MSK and WU) independently screened the abstracts to compile a list of the top 100 most cited CMR articles. In cases of discrepancy between the reviewers, consensus was achieved with the help of a third independent reviewer (IBR). For each article, citation count, first, intermediate and senior author, country of origin, year of publication, number of authors and journal name along with its impact factor were extracted.

The relationship between the impact factor of a journal and the number of top 100 cited articles was analyzed using the Pearson product moment correlation co-efficient. All data are presented in the form of median and inter-quartiles (IQ). For all cases, a *P-*value of less than 0.05 was considered significant.

## Results

**Fig. 1:**
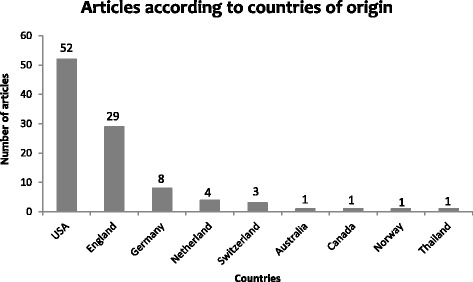
The 100 top-cited Cardiovascular Magnetic Resonance articles classified with respect to country of origin

**Fig. 2:**
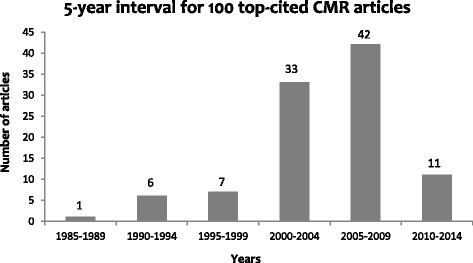
The 100 top-cited Cardiovascular Magnetic Resonance articles quantized by 5-year intervals

Additional file 1: Table S1 shows the list of top 100 CMR articles. The median number of citations was 319.5 with a range of 178 to 1925. Only 17 articles were cited more than 500 times with the majority (*n =* 72) of the articles being cited between 200–499 times. There were 9 different countries of origin for the top 100 cited articles (Fig. 1). More than half of the articles (*n =* 52) were from the United States of America, and more than one-quarter (*n =* 29) from the United Kingdom. The vast majority (*n =* 86) of articles were published between 2000–2014. Only one article was published before 1990. The 5-year interval with the highest number (*n =* 42) of studies was 2005–2009 (Fig. 2).

*Circulation* and *Journal of the American College of Cardiology* published nearly 2/3rds (*n =* 62) of the list of top 100 CMR articles (Table 1). *Journal of Cardiovascular Magnetic Resonance* and *European Heart Journal each* contributed 8 and 7 articles to the list, respectively. All the other journals had less than 5 studies each. General medical journals such as *The Lancet* and *The New England Journal of Medicine* had 2 and 4 articles in the list respectively. Within the list, we found a statistically significant correlation between the number of top-cited articles and journal impact factor (*P <* 0.005).

**Table 1:** Top-cited articles according to journal and their impact factors (only journals with 2 or more articles have been shown)

Journal’s Name	Number of Articles	2015 Impact Factor
*Circulation*	34	15.07
*Journal of the American College of Cardiology*	28	16.50
*Journal of Cardiovascular Magnetic Resonance*	8	4.72
*European Heart Journal*	7	15.20
*Radiology*	4	6.87
*New England Journal of Medicine*	4	55.87
*Magnetic Resonance in Medicine*	2	3.57
*IEEE Transactions on Medical Imaging*	2	3.39
*Heart*	2	5.59
*Lancet*	2	45.21

We found 10 authors who had greater than 5 publications in the list. These included Manning (*n =* 22), Pennell (*n =* 17), and Moon (*n =* 12) with at least 10 each (Table 2). The key study areas in the CMR articles were mainly cardiomyopathy (*n =* 18), coronary artery disease (*n =* 9),myocardial infarction(*n =* 11), myocardial anatomy (*n =* 7) and myocardial fibrosis (*n =* 6).

**Table 2:** Authors with ≥ 5 articles in the top 100 articles

	Author Position in the Article				
Author	Number of articles in list	First	Last	Other	Affiliation(s)
Manning, W.J	22	2	9	11	Beth Israel Deaconess Medical Center, Boston, MA
Pennell, D.J	17	1	14	2	Royal Brompton Hospital, Imperial College London, UK
Moon, J.C	12	5	1	6	Barts Heart Hospital Imaging Centre, London, UK
Bluemke, D.A.,	9	1	0	8	Johns Hopkins University, Baltimore, MD; Radiology and Imaging Sciences, NIH Clinical Center, Bethesda, MD
Rene M Botnar	8	3	2	3	Beth Israel Deaconess Medical Center; King’s College, London, UK
Mathias Stuber	7	1	0	6	Beth Israel Deaconess Medical Center, Boston, MA; Johns Hopkins University, Baltimore, MD; University of Lausanne, Switzerland
Kraig V Kissinger	7	0	0	7	Beth Israel Deaconess Medical Center, Boston, MA
Hundley, W.G.,	6	3	0	3	Forest Baptist Medical Center, Winston-Salem, NC
Lima, J.A.C	6	0	4	2	Johns Hopkins University, Baltimore, MD
Arai, A.E	6	0	3	3	National Heart, Lung, and Blood Institute, Bethesda, MD
Kim R.J	5	1	3	1	Duke University, Durham, NC
McVeigh, E.R	5	0	1	4	Johns Hopkins University, Baltimore, MD; University of California at San Diego, San Diego, CA; National Institutes of Health, Bethesda,
Judd R.M	5	0	2	3	Weil Cornell Medical College, New York, NY
Mathias G Friedrich	5	1	1	3	Cardiovascular Institute of Alberta, Calgary, Canada; McGill University, Montreal, Canada

## Discussion

In our study to identify the top 100 highly-cited CMR articles, we found that the majority (*n =* 86) of the top cited articles were published between the time period 2000–2014. This is in contrast with bibliometrics published in other fields such as orthopedics [13], neurosurgery [11] and general surgery [9] where the peak time period for top cited articles was 1965 to 1980. However, a general cardiology bibliometric [7] had a similar peak time period 2001–2010. This suggests that the field of the cardiology as a whole is evolving rapidly and in sync with CMR. It also argues against the theory, which has been previously stated in other bibliometrics [14–16] that the article’s true value cannot be judged correctly till at least 3 decades post-publication. For our CMR bibliometric, the peak time period of 2000–2014 is also not surprising considering that evolution in technology which has resulted in novel CMR applications facilitating early and definitive detection of various cardiovascular diseases. When considering the time period for bibliometrics, there are two important factors to consider. Firstly, the obliteration by incorporation phenomenon [17] which states that landmark articles are sometimes cited rarely because the information they provide becomes so widely used and embedded in the daily practice of each clinician, that researchers do not feel the need to cite that particular study. Obliteration by incorporation phenomenon sometimes leads to recent peak time periods of bibliometrics. Secondly, the inherent bias of bibliometrics against recent papers might lead to some extremely important papers not being included in such an analysis as it takes time to accumulate citations [18].

We observed that the majority (*n =* 62) of the top cited CMR articles were published in high impact factor cardiology journals such as *Circulation* and the *Journal of the American College of Cardiology*. It shows that cardiovascular imaging researchers tend to publish important studies in influential cardiology journals rather than radiology or cardiovascular imaging journals. This might be due to the fact that radiology or even cardiovascular imaging journals do not have very high impact factors. Only 6 articles were published in the top-tier general medical journals such as *The Lancet* and *The New England Journal of Medicine*. This is not surprising as cardiovascular imaging papers are normally not of interest to a general medical audience and thus larger outcome studies are needed in CMR. Overall, the trend of CMR bibliometrics seems to follow the Bradford’s law, a concept suggested by Brookes [19] that most researchers get their citations from a few specific core journals. When authors deviate from these journals, the impact of their article is reduced and thus most researchers try to stay with those few specific journals. In this instance, those few core journals were *Circulation* and *Journal of American College of Cardiology*.

We also found that the majority of the top-cited CMR studies focused on various sub topics ranging from evaluation of various adult and congenital heart diseases to assessment of myocardial anatomy and cardiac volumes in heart failure. Knowing the different areas of study in the top 100 list of CMR articles is vital because it can have important implications not only for editors and stakeholders in selecting and judging future scientific work but also for young scientists to publish effectively in the future. In our top-100 list, the top areas of study were evaluation of coronary artery disease, cardiomyopathies and myocardial infarction/fibrosis. Only a handful of studies on congenital and valvular heart disease made it to the list. Such trends are not surprising as coronary artery disease is one of the overall leading causes of mortality and morbidity. As with previous cardiology bibliometrics, congenital heart diseases are underrepresented. Considering that the burden of congenital heart disease is projected to rise [20], future bibliometric analyses may identify more papers on congenital heart diseases and other topics such as valvular heart diseases, areas in which only a few CMR landmark studies have been published to date. Interestingly, only 3 three articles from our top 100 list were present in the most recent cardiology bibliometric analysis published [7].

Several interesting observations can be made in regard to the list of the authors who we feel have had a great impact on the field of CMR. We identified 14 authors who had 5 or more citations in the list of top 100 CMR articles. This is a larger number in comparison with other bibliometrics. For instance emergency medicine [6] and dermatology [16] citation classics had only 5 and 6 authors respectively who had 4 or more citations in the top 100 list. It shows that there a group of eminent cardiologists/radiologists who are publishing most of the influential CMR studies. It is important to consider that scientists who frequently produce high quality work have a higher chance of academic promotion, and editors are more likely to accept their work and invite them to review articles [21].

Overall, it was seen that the majority (*n =* 81) of the articles came from the United States and the United Kingdom. This is consistent with other bibliometrics lists where the United States contributed most of the articles. As expected, the median number of citations in CMR was much lower than the other fields such as emergency medicine, radiology, neurosurgery, cardiology and respiratory medicine. Currently there is no bibliometric data on other cardiovascular imaging tests that could be used for better comparison in the case of CMR.

There are several limitations that should be considered. Firstly the effect of incomplete citation has not been taken in to account. Incomplete citation [10] is the method of taking summarized conclusions from systematic reviews rather than looking at each article individually. Secondly, the impact of self-citation was also not considered. Self-citation has not been shown to have a major impact on bibliometric measures [22], especially over a long duration; however, it is crucial to study its impact on future CMR bibliometrics because in the current analysis more than 10 authors had 5 or more publications in the top 100 list indicating some research collaboration. Thirdly, it has been reported that Scopus tends to miss older citations resulting in omission of studies before 1980 [23, 24]. Moreover, textbooks were not included in our methodology which could have led to some omission bias. However, considering that CMR is a relatively new technique, we doubt that it would have had a major impact on our findings.

## Conclusion

In summary, we identified and analyzed the characteristics of the 100 most-cited articles in CMR. Such analyses may provide help guide researchers and funding agencies on the most important research areas in the field.

## Additional file

Additional file 1: List of top 100 citations. (DOCX 26 kb)

## Abbreviations

CMR: Cardiovascular Magnetic Resonance
WOS: Web of Science
