# Supplementary material for: Top 100 cited articles in cardiovascular magnetic resonance: a bibliometric analysis
Source: J Cardiovasc Magn Reson. 2016 Nov 21;18:87. doi: 10.1186/s12968-016-0303-9 (PMC5116819; doi:10.1186/s12968-016-0303-9)
Supplement: Additional file 1: — List of top 100 citations. (DOCX 26 kb) [file 12968_2016_303_MOESM1_ESM.docx]

| S.NO | Title | Number of Citations |
| --- | --- | --- |
| 1 | The use of contrast-enhanced magnetic resonance imaging to identify reversible myocardial dysfunction | 1925 |
| 2 | Coronary magnetic resonance angiography for the detection of coronary stenoses | 967 |
| 3 | Prognostic significance of microvascular obstruction by magnetic resonance imaging in patients with acute myocardial infarction | 957 |
| 4 | ACCF/ACR/SCCT/SCMR/ASNC/NASCI/SCAI/SIR 2006 Appropriateness Criteria for Cardiac Computed Tomography and Cardiac Magnetic Resonance Imaging* * Developed in accordance with the principles and methodology outlined by ACCF | 862 |
| 5 | Contrast-enhanced MRI and routine single photon emission computed tomography (SPECT) perfusion imaging for detection of subendocardial myocardial infarcts: An imaging study | 847 |
| 6 | Clinical indications for cardiovascular magnetic resonance (CMR): Consensus Panel report | 812 |
| 7 | Cardiovascular T2-star (T2*) magnetic resonance for the early diagnosis of myocardial iron overload | 812 |
| 8 | Cardiovascular Magnetic Resonance in Myocarditis: A JACC White Paper | 799 |
| 9 | Comparison of left ventricular ejection fraction and volumes in heart failure by echocardiography, radionuclide ventriculography and cardiovascular magnetic resonance. Are they interchangeable? | 665 |
| 10 | Cardiovascular Magnetic Resonance Assessment of Human Myocarditis: A Comparison to Histology and Molecular Pathology | 638 |
| 11 | Comparison of interstudy reproducibility of cardiovascular magnetic resonance with two-dimensional echocardiography in normal subjects and in patients with heart failure or left ventricular hypertrophy | 631 |
| 12 | Differentiation of heart failure related to dilated cardiomyopathy and coronary artery disease using gadolinium-enhanced cardiovascular magnetic resonance | 620 |
| 13 | A preliminary report comparing magnetic resonance coronary angiography with conventional angiography | 610 |
| 14 | Cardiovascular Magnetic Resonance, Fibrosis, and Prognosis in Dilated Cardiomyopathy | 547 |
| 15 | Toward clinical risk assessment in hypertrophic cardiomyopathy with gadolinium cardiovascular magnetic resonance | 537 |
| 16 | Normal Human Right and Left Ventricular Mass, Systolic Function, and Gender Differences by Cine Magnetic Resonance Imaging | 507 |
| 17 | Assessment of Myocardial Perfusion in Coronary Artery Disease by Magnetic Resonance: A Comparison With Positron Emission Tomography and Coronary Angiography | 505 |
| 18 | Abnormal subendocardial perfusion in cardiac syndrome X detected by cardiovascular magnetic resonance imaging | 491 |
| 19 | Occurrence and frequency of arrhythmias in hypertrophic cardiomyopathy in relation to delayed enhancement on cardiovascular magnetic resonance | 472 |
| 20 | Left ventricular non-compaction: Insights from cardiovascular magnetic resonance imaging | 455 |
| 21 | Noninvasive coronary artery imaging magnetic resonance angiography and multidetector computed tomography angiography: a scientific statement from the American Heart Association Committee on Cardiovascular Imaging and Intervention of the Council on Cardiovascular Radiology and Intervention, and the Councils on Clinical Cardiology and Cardiovascular Disease in the Young | 447 |
| 22 | Cardiovascular magnetic resonance in cardiac amyloidosis | 438 |
| 23 | Diagnostic performance of cardiovascular magnetic resonance in patients with suspected acute myocarditis: Comparison of different approaches | 435 |
| 24 | ACCF/ACR/AHA/NASCI/SCMR 2010 expert consensus document on cardiovascular magnetic resonance: a report of the American College of Cardiology Foundation Task Force on Expert Consensus Documents | 432 |
| 25 | Normal human left and right ventricular dimensions for MRI as assessed by turbo gradient echo and steady-state free precession imaging sequences | 432 |
| 26 | Characterization of the peri-infarct zone by contrast-enhanced cardiac magnetic resonance imaging is a powerful predictor of post-myocardial infarction mortality | 427 |
| 27 | Improved coronary artery definition with T2-weighted, free-breathing, three-dimensional coronary MRA | 421 |
| 28 | Noninvasive detection of myocardial ischemia from perfusion reserve based on cardiovascular magnetic resonance | 418 |
| 29 | Coronary arteries: breath-hold MR angiography. | 406 |
| 30 | Right ventricular function in adults with repaired tetralogy of Fallot assessed with cardiovascular magnetic resonance imaging: Detrimental role of right ventricular outflow aneurysms or akinesia and adverse right-to-left ventricular interaction | 406 |
| 31 | Reduction in sample size for studies of remodeling in heart failure by the use of cardiovascular magnetic resonance | 405 |
| 32 | Infarct tissue heterogeneity by magnetic resonance imaging identifies enhanced cardiac arrhythmia susceptibility in patients with left ventricular dysfunction | 404 |
| 33 | Interstudy reproducibility of right ventricular volumes, function, and mass with cardiovascular magnetic resonance | 392 |
| 34 | Delayed enhancement cardiovascular magnetic resonance assessment of non-ischaemic cardiomyopathies | 383 |
| 35 | The histologic basis of late gadolinium enhancement cardiovascular magnetic resonance in hypertrophic cardiomyopathy | 382 |
| 36 | Impact of unrecognized myocardial scar detected by cardiac magnetic resonance imaging on event-free survival in patients presenting with signs or symptoms of coronary artery disease | 380 |
| 37 | Phase contrast cine magnetic resonance imaging. | 369 |
| 38 | Evaluation of Diffuse Myocardial Fibrosis in Heart Failure With Cardiac Magnetic Resonance Contrast-Enhanced T1 Mapping | 358 |
| 39 | Preoperative thresholds for pulmonary valve replacement in patients with corrected tetralogy of Fallot using cardiovascular magnetic resonance | 358 |
| 40 | MR-IMPACT: comparison of perfusion-cardiac magnetic resonance with single-photon emission computed tomography for the detection of coronary artery disease in a multicentre, multivendor, randomized trial | 352 |
| 41 | Noninvasive coronary vessel wall and plaque imaging with magnetic resonance imaging | 350 |
| 42 | Double-oblique free-breathing high resolution three-dimensional coronary magnetic resonance angiography | 347 |
| 43 | Retrospective determination of the area at risk for reperfused acute myocardial infarction with T2-weighted cardiac magnetic resonance imaging: Histopathological and displacement encoding with stimulated echoes (DENSE) functional validations | 347 |
| 44 | Prognostic value of cardiac magnetic resonance stress tests: Adenosine stress perfusion and dobutamine stress wall motion imaging | 343 |
| 45 | Serial cardiac magnetic resonance imaging of injected mesenchymal stem cells | 340 |
| 46 | Sequelae of acute myocardial infarction regarding cardiac structure and function and their prognostic significance as assessed by magnetic resonance imaging | 331 |
| 47 | Delayed enhancement and T2-weighted cardiovascular magnetic resonance imaging differentiate acute from chronic myocardial infarction | 328 |
| 48 | Blood flow imaging by cine magnetic resonance | 327 |
| 49 | Identification of anomalous coronary arteries and their anatomic course by magnetic resonance coronary angiography | 325 |
| 50 | First-pass nuclear magnetic resonance imaging studies using gadolinium-DTPA in patients with coronary artery disease | 323 |
| 51 | Diagnostic Performance of Stress Cardiac Magnetic Resonance Imaging in the Detection of Coronary Artery Disease. A Meta-Analysis | 316 |
| 52 | Cardiovascular magnetic resonance and single-photon emission computed tomography for diagnosis of coronary heart disease (CE-MARC): A prospective trial | 314 |
| 53 | Right ventricular function in adults with repaired tetralogy of Fallot assessed with cardiovascular magnetic resonance imaging: Detrimental role of right ventricular outflow aneurysms or akinesia and adverse right-to-left ventricular interaction | 307 |
| 54 | Equilibrium contrast cardiovascular magnetic resonance for the measurement of diffuse myocardial fibrosis: Preliminary validation in humans | 302 |
| 55 | Three-dimensional black-blood cardiac magnetic resonance coronary vessel wall imaging detects positive arterial remodeling in patients with nonsignificant coronary artery disease | 300 |
| 56 | Normalized Left Ventricular Systolic and Diastolic Function by Steady State Free Precession Cardiovascular Magnetic Resonance | 298 |
| 57 | Unaliasing by Fourier-encoding the overlaps using the temporal dimension (UNFOLD), applied to cardiac imaging and fMRI | 295 |
| 58 | Utility of cardiac magnetic resonance imaging in the diagnosis of hypertrophic cardiomyopathy | 278 |
| 59 | Standardized cardiovascular magnetic resonance imaging (CMR) protocols, society for cardiovascular magnetic resonance: board of trustees task force on standardized protocols | 278 |
| 60 | Safety of Magnetic Resonance Imaging in Patients With Cardiovascular Devices An American Heart Association Scientific Statement From the Committee on Diagnostic and Interventional Cardiac Catheterization, Council on Clinical Cardiology, and the Council on Cardiovascular Radiology and Intervention: Endorsed by the American College of Cardiology Foundation, the North American Society for Cardiac Imaging, and the Society for Cardiovascular Magnetic Resonance | 269 |
| 61 | Imaging heart motion using harmonic phase MRI | 269 |
| 62 | Myocardial scar visualized by cardiovascular magnetic resonance imaging predicts major adverse events in patients with hypertrophic cardiomyopathy | 269 |
| 63 | Magnetic resonance assessment of the substrate for inducible ventricular tachycardia in nonischemic cardiomyopathy | 269 |
| 64 | Late Gadolinium Enhancement by Cardiovascular Magnetic Resonance Heralds an Adverse Prognosis in Nonischemic Cardiomyopathy | 267 |
| 65 | In vivo molecular imaging of acute and subacute thrombosis using a fibrin-binding magnetic resonance imaging contrast agent | 267 |
| 66 | A Randomized, Placebo-Controlled, Double-Blind Trial of the Effect of Combined Therapy With Deferoxamine and Deferiprone on Myocardial Iron in Thalassemia Major Using Cardiovascular Magnetic Resonance | 265 |
| 67 | Clinical Characteristics and Cardiovascular Magnetic Resonance Findings in Stress (Takotsubo) Cardiomyopathy | 264 |
| 68 | Evaluation of the accuracy of gadolinium-enhanced cardiovascular magnetic resonance in the diagnosis of cardiac sarcoidosis | 263 |
| 69 | Ventricular fibrosis suggested by cardiovascular magnetic resonance in adults with repaired tetralogy of Fallot and its relationship to adverse markers of clinical outcome | 263 |
| 70 | ACCF/AHA clinical competence statement on cardiac imaging with computed tomography and magnetic resonance: a report of the American College of Cardiology Foundation/American Heart Association/American College of Physicians Task Force on Clinical Competence and Training | 262 |
| 71 | Utility of fast cine magnetic resonance imaging and display for the detection of myocardial ischemia in patients not well suited for second harmonic stress echocardiography | 258 |
| 72 | Detecting acute coronary syndrome in the emergency department with cardiac magnetic resonance imaging | 258 |
| 73 | Improved Detection of Coronary Artery Disease by Stress Perfusion Cardiovascular Magnetic Resonance With the Use of Delayed Enhancement Infarction Imaging | 258 |
| 74 | Magnetic resonance imaging to assess the hemodynamic effects of pulmonary valve replacement in adults late after repair of tetralogy of Fallot | 256 |
| 75 | Myocardial iron clearance during reversal of siderotic cardiomyopathy with intravenous desferrioxamine: A prospective study using T2* cardiovascular magnetic resonance | 241 |
| 76 | Importance of imaging method over imaging modality in noninvasive determination of left ventricular volumes and ejection fraction: assessment by two-and three-dimensional echocardiography and magnetic resonance imaging | 240 |
| 77 | Gadolinium enhanced cardiovascular magnetic resonance in Anderson-Fabry disease | 235 |
| 78 | Hypertrophic cardiomyopathy phenotype revisited after 50 years with cardiovascular magnetic resonance | 228 |
| 79 | Compressed sensing in dynamic MRI | 227 |
| 80 | Cardiac T2* magnetic resonance for prediction of cardiac complications in Thalassemia Major | 225 |
| 81 | Reference right ventricular systolic and diastolic function normalized to age, gender and body surface area from steady-state free precession cardiovascular magnetic resonance | 223 |
| 82 | Assessment of myocardial fibrosis with cardiovascular magnetic resonance | 221 |
| 83 | A single breath-hold multiecho T2* cardiovascular magnetic resonance technique for diagnosis of myocardial iron overload | 217 |
| 84 | Gadolinium delayed enhancement cardiovascular magnetic resonance correlates with clinical measures of myocardial infarction | 210 |
| 85 | Detection of apical hypertrophic cardiomyopathy by cardiovascular magnetic resonance in patients with non-diagnostic echocardiography | 210 |
| 86 | Three-dimensional black-blood cardiac magnetic resonance coronary vessel wall imaging detects positive arterial remodeling in patients with nonsignificant coronary artery disease | 210 |
| 87 | Prognosis of negative adenosine stress magnetic resonance in patients presenting to an emergency department with chest pain | 206 |
| 88 | Ventricular size and function assessed by cardiac MRI predict major adverse clinical outcomes late after tetralogy of Fallot repair | 206 |
| 89 | Infarct size by contrast enhanced cardiac magnetic resonance is a stronger predictor of outcomes than left ventricular ejection fraction or end-systolic volume index: prospective cohort study | 205 |
| 90 | Gadolinium Cardiovascular Magnetic Resonance Predicts Reversible Myocardial Dysfunction and Remodeling in Patients With Heart Failure Undergoing β-Blocker Therapy | 205 |
| 91 | The role of cardiovascular magnetic resonance in patients presenting with chest pain, raised troponin, and unobstructed coronary arteries | 195 |
| 92 | Magnetic resonance imaging determination of cardiac prognosis | 196 |
| 93 | Prognostic Significance and Determinants of Myocardial Salvage Assessed by Cardiovascular Magnetic Resonance in Acute Reperfused Myocardial Infarction | 195 |
| 94 | Value of Delayed-Enhancement Cardiovascular Magnetic Resonance Imaging in Predicting Myocardial Viability After Surgical Revascularization | 194 |
| 95 | Improved survival of thalassaemia major in the UK and relation to T2* cardiovascular magnetic resonance | 192 |
| 96 | Breath-hold FLASH and FISP cardiovascular MR imaging: Left ventricular volume differences and reproducibility | 190 |
| 97 | Tag and Contour Detection in Tagged MR Images of the Left Ventricle | 190 |
| 98 | Clinical utility and safety of a protocol for noncardiac and cardiac magnetic resonance imaging of patients with permanent pacemakers and implantable-cardioverter defibrillators at 1.5 tesla | 188 |
| 99 | Accurate and reproducible measurement of left ventricular volume and ejection fraction by contrast echocardiography: A comparison with magnetic resonance imaging | 182 |
| 100 | Cardiovascular Magnetic Resonance in Clinically Suspected Cardiac Amyloidosis. Noninvasive Imaging Compared to Endomyocardial Biopsy | 178 |
